# Copper Enhanced Monooxygenase Activity and FT-IR Spectroscopic Characterisation of Biotransformation Products in Trichloroethylene Degrading Bacterium: *Stenotrophomonas maltophilia* PM102

**DOI:** 10.1155/2013/723680

**Published:** 2013-09-08

**Authors:** Piyali Mukherjee, Pranab Roy

**Affiliations:** ^1^Department of Biotechnology, Burdwan University, Golapbag, Burdwan, West Bengal, 713104, India; ^2^Department of Biotechnology, Haldia Institute of Technology, Haldia, West Bengal, 721657, India

## Abstract

*Stenotrophomonas maltophilia* PM102 (NCBI GenBank Acc. no. JQ797560) is capable of growth on trichloroethylene as the sole carbon source. In this paper, we report the purification and characterisation of oxygenase present in the PM102 isolate. Enzyme activity was found to be induced 10.3-fold in presence of 0.7 mM copper with a further increment to 14.96-fold in presence of 0.05 mM NADH. Optimum temperature for oxygenase activity was recorded at 36°C. The reported enzyme was found to have enhanced activity at pH 5 and pH 8, indicating presence of two isoforms. Maximum activity was seen on incubation with benzene compared to other substrates like TCE, chloroform, toluene, hexane, and petroleum benzene. *K*_*m*_ and *V*_max_ for benzene were 3.8 mM and 340 U/mg/min and those for TCE were 2.1 mM and 170 U/mg/min. The crude enzyme was partially purified by ammonium sulphate precipitation followed by dialysis. Zymogram analysis revealed two isoforms in the 70% purified enzyme fraction. The activity stain was more prominent when the native gel was incubated in benzene as substrate in comparison to TCE. Crude enzyme and purified enzyme fractions were assayed for TCE degradation by the Fujiwara test. TCE biotransformation products were analysed by FT-IR spectroscopy.

## 1. Introduction 

**Figure 1:**
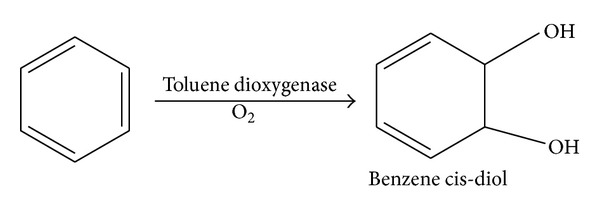
Oxidation of benzene to benzene *cis*-diol by toluene dioxygenase.

Hydrocarbon dioxygenases have broad substrate specificity which makes them attractive candidates for production of industrially and medically important chemicals and development of bioremediation technology. Bacterial aromatic oxygenases are multicomponent enzymes that add oxygen molecules to the aromatic nucleus to form arene cis-diols. For example, toluene dioxygenase that catalyses the following reaction (Figure 1) [1].

Toluene 4-monooxygenase (T4MO) of *Pseudomonas mendocina* KR1, toluene 3-monooxygenase (T3MO) of *Ralstonia pickettii* PKO1, and toluene ortho-monooxygenase (TOM) of *Burkholderia cepacia* G4 convert benzene to phenol, catechol, and 1,2,3-trihydroxybenzene by successive hydroxylations [2].

Current interests in structure and function of dioxygenases are due to their role in bioremediation of contaminants from our environment and in the green synthesis of cis-diols that have significant industrial and medical importance such as development of polyphenylene and prostaglandin E2*α* [2–5]. Crixivan (indinavir) is an orally active HIV protease inhibitor. A key intermediate in its synthesis is (-)-*cis*-(1*S*,2*R*)-1-aminoindan-2-ol. This compound can be synthesized directly from enantiopure *cis*-(1*S*,2*R*)-dihydroxyindan. Toluene dioxygenase oxidizes indene to produce the desired enantiomer [6]. 

**Figure 2:**
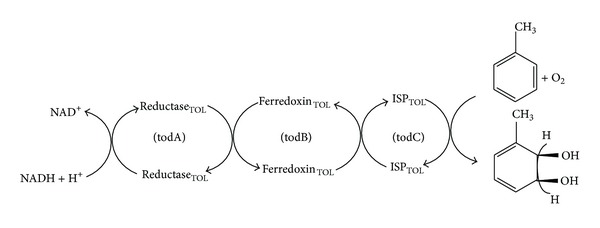
Oxidation of toluene to cis-toluene dihydrol by toluene dioxygenase. Electrons are transferred from NADH through a flavoprotein reductase (reductase_TOL_) to a Rieske (2Fe-2S) protein (ferredoxin_TOL_). The latter reduces the oxygen component, an iron-sulfur protein (ISP_TOL_), which, in the presence of exogenous ferrous ion, catalyses the stereospecific addition of dioxygen to the aromatic nucleus.

All members of the dioxygenase family have one or two electron transport proteins preceding their oxygenase components, as shown in Figure 2 for toluene dioxygenase system [7]. 

*Pseudomonas cepacia *G4 was the first organism to degrade TCE, reported to contain toluene monooxygenase. Toluene monooxygenase and dioxygenase [8, 9]; methane monooxygenase [10]; propane monooxygenase [11]; ammonia monooxygenase [12]; and also mammalian liver cytochrome P450 monooxygenase have been implicated in TCE degradation. In 1996, Werlen et al. [13] identified four dioxygenase families (naphthalene, toluene/benzene, biphenyl, and benzoate/toluate) based on amino acid sequence comparisons of the catalytic oxygenase *α* subunits. A novel tce350 gene product from *Stenotrophomonas maltophilia* PM102 was shown to belong to the dioxygenase family of proteins due to the presence of 71.6% each of alternating *α* helix and *β* strand [14]. Monooxygenases can be classified according to the cofactors required for catalytic activity. Heme-dependent monooxygenases are cytochrome P450 monooxygenases, for example, CYP102 from *Bacillus megaterium* BM3 that hydroxylates a variety of alkanes, fatty acids, and aromatic compounds [15]. In all CYPs, a strictly conserved cysteine is found in the active site that acts as the fifth ligand of the heme-iron center, thereby activating the metal complex [16]. CYPs catalyze a wide variety of oxidations. Besides epoxidations and hydroxylations, these monooxygenases are also able to perform heteroatom dealkylations and oxidations, oxidative deaminations, dehalogenations, dehydrogenations, dehydrations, and reductions [17]. Flavin-dependent monooxygenases require FAD/FMN, for example, luciferases and Type II Baeyer-Villiger monooxygenases [18]. Copper-dependent monooxygenases constitute a relatively small family of enzymes that require copper ions for hydroxylation of their substrates. An example of these monooxygenases is the membrane-associated methane monooxygenase (pMMO) from *Methylococcus capsulatus*. It has been reported that this enzyme contains up to 20 copper ions per heterotrimer, in which the ions are involved in either activation of molecular oxygen or electron transfer [19]. Nonheme iron-dependent monooxygenases utilize two iron atoms as cofactor for their oxidative activity. The best-characterized member of this family is the soluble methane monooxygenase (sMMO) from *M. capsulatus* [20]. Other members of this family are alkene monooxygenases, phenol hydroxylase, and toluene-4-monooxygenase. Another family of monooxygenases is the pterin-dependent monooxygenases, mainly of eukaryotic origin, that hydroxylate the amino acids phenylalanine, tyrosine, and tryptophan at their aromatic ring.

 In this communication, we present the biochemical characterisation of a novel oxygenase induced by copper and benzene from PM102 isolate that utilises trichloroethylene (TCE) as the sole carbon source. A simple spectrophotometric method was used to measure enzyme activity [21]. Purification of the enzyme and investigation of its probable role in TCE degradation have been documented. Optimisation of protocol for activity staining is also reported. Fourier transform infrared (FT-IR) spectroscopy has become an important tool for rapid analysis of complex biological samples. The infrared absorbance spectrum could be regarded as a “fingerprint,” which is a characteristic of biochemical substances. The ability to use FT-IR to rapidly distinguish between biotransformation product mixtures suggests this approach might be a valuable tool for screening large biotransformation assays for novel products [22]. Determination of enzymatic breakdown products by the PM102 isolate through FT-IR spectroscopy has been attempted in this communication.

## 2. Materials and Methods 

### 2.1. Strain and Culture Conditions

*Stenotrophomonas maltophilia* PM102 was isolated in our laboratory from soil samples obtained from Asansol and Dhanbad industrial belt, India. It was identified by 16S rDNA sequencing (GenBank Acc. No. JQ797560). The PM102 isolate could grow on TCE as the sole carbon source and degraded TCE efficiently [23]. TCE-induced proteins from the PM102 isolate were previously identified [24], and TCE degradation in presence of other organic pollutants was also documented [25]. The PM102 isolate was grown in minimal medium: Na_2_HPO_4_—1 g/L, K_2_HPO_4_—3 g/L, NH_4_Cl—1 g/L, and MgSO_4_·7H_2_O—0.4 g/L with 0.1% peptone and 0.2% of different carbon sources: TCE, toluene, chloroform, benzene, hexane, and petroleum benzene.

### 2.2. Crude Enzyme Extract Preparation

PM102 cells were grown in a shaker incubator (150 rpm), at 34°C, for 24 hours in 200 mL minimal medium with 0.2% of the different carbon sources and 0.1% peptone. Cells were harvested by centrifugation at 10,000 rpm for 10 minutes at 4°C, and cell pellet was suspended in 1.5 mL solution I (10 mM EDTA pH 8, 50 mM glucose, and 25 mM tris HCl pH 8) with 100 *μ*L 10 mg/mL lysozyme. The suspension was vortexed and kept at 4°C for 15 minutes followed by temperature shock at 37°C for 1 hour and further incubated at 4°C for 30 minutes. The suspension was centrifuged at 5000 rpm for 5 minutes at 4°C and the supernatant was stored as crude enzyme extract at −20°C for further studies. The supernatant of the cell cultured in media with different carbon sources was also used as the crude enzyme for mono-oxygenase assay.

### 2.3. Oxygenase Assay

10 mL reaction mixture contained 5 mL reactant A (25 mg o-Dianisidine + 1 mL methanol + 49 mL phosphate buffer), 1.5 mL phosphate buffer (0.1 M KH_2_PO_4_, pH 5), 3.5 mL substrate—TCE and other organic compounds, 100 *μ*L (90 *μ*g crude enzyme extract intracellular), and 0.05 mM NADH. For extracellular assay, 1 mL of the cell-free medium supernatant (16 *μ*g protein) was added as crude enzyme extract. Protein content in cell lysate and sup was measured by Bradford assay [26]. A dark red colour was formed in the substrate phase only which was pipetted out, and absorbance was noted at 480 nm. The basic principle is based on the property of oxygenases to use molecular oxygen to oxidize the hydrocarbon-substrate molecules by means of an electron transport system. Ortho-dianisidine (ortho-DNS) acts as a chromogenic acceptor of oxygen.

### 2.4. Characterisation of the Oxygenase

Effect of various metal cofactors was studied by adding 1 mM of the following metal salts in 10 mL reaction mixture: copper sulphate, ferric chloride, cobalt bromide, manganese sulfate, zinc chloride, aluminium chloride, lead nitrate, silver nitrate, calcium chloride, magnesium sulphate, sodium chloride, and potassium dihydrogen phosphate. Optimum copper concentration for enzymatic activity was determined by varying the concentration of copper sulfate added to 10 mL reaction mixture from 0.05 mM to 5 mM.

Optimum NADH concentration was determined by varying NADH concentration from 0.01 mM to 2 mM in presence of 0.7 mM Cu. Substrate specificity was analysed with TCE, benzene, toluene, chloroform, hexane and petroleum benzine separately to 10 mL reaction mixture setup each. *K*_*m*_ and *V*_max⁡_ were calculated by varying concentrations of benzene and TCE under fixed enzyme concentration. Effect of temperature was studied by setting up the reaction condition at 4, 20, 30, 36, and 42°C, respectively. Optimisation of pH for oxygenase activity was performed by varying the pH of the phosphate buffer used in the reaction mixture from pH 3 to pH 8.5, varying at intervals of 0.5. For pH 8.5 to pH 10, glycine-NaOH buffer was used. Activity in lysate (intracellular) and supernatant (extracellular) was also studied. 

### 2.5. Native PAGE for Zymogram Analysis

For zymogram studies, PM102 cells were grown in minimal medium with 0.2% TCE for 24 hours followed by induction with 0.2% of various carbon sources: TCE, toluene, chloroform, benzene, hexane, and petroleum for another 24 hours. Intracellular proteins were extracted by lysozyme, and protein concentration was measured by the Bradford assay. About 100 *μ*g of proteins was resolved by 8% native gel that was incubated in 10 mL reactant A, 9 mL 0.1 M phosphate buffer (pH 5), 1 mL substrate (benzene/TCE), and 0.7 mM copper. 

### 2.6. Enzyme Purification

To understand which particular protein band gave colour in activity staining, purification of the crude enzyme extract was done by ammonium sulphate precipitation followed by dialysis. As maximum enzyme activity was recorded in the supernatant, salt precipitation was carried out with 200 mL culture supernatant of PM102 grown in 0.2% TCE with 0.2% benzene induction. The online program ammonium sulphate calculator from EnCor Biotechnology, Inc, Gainesville, Florida [28], was used. Precipitation was done at 10% and 30% for 2-3 hours and at 50%, 70%, and 90% of ammonium sulphate for overnight incubation, with magnetic stirrer at 4°C. After each precipitation step, the fraction was centrifuged at 12,000 rpm for 15 minutes. Precipitate obtained as pellet (if any) was dissolved in 500 *μ*L of 10 mM PBS for removal of Ammonium sulphate by dialysis against 200 mL 10 mM PBS for 2 hours at 25°C, followed by another 2 hours after buffer change. A third buffer change was given for overnight dialysis at 4°C. The fractions after dialysis were resolved through 8% native gel. One-half of the gel was stained with Coomassie blue, and the other half was subjected to activity staining.

### 2.7. Investigating TCE Degradation Activity of the Purified Enzyme Extract

TCE degradation studies with the unpurified crude protein extract and with the purified enzyme fractions (extracellular) were carried out by the Fujiwara test. In this reaction, trichloroethylene in presence of pyridine and caustic alkali gives a red colour compound [29]. In 20 mL of 10 mM PBS, 100 *μ*L of enzyme extract was added and incubated with 0.2% TCE added initially. 2 mL aliquots were drawn after respective time intervals, that is, 1, 2, 3, and 24 hours, and Fujiwara assay was done. Absorbance of the red upper aqueous phase was measured at 470 nm. As TCE gets degraded with time, the intensity of the red colour decreases with a corresponding decline in O.D. The experiment was carried out in four sets with crude unpurified enzyme, 30% Ammonium sulphate fraction, 50% fraction and 70% fraction of the extract. 

### 2.8. FT-IR Analysis to Determine Biodegradation Products

Biomass obtained with TCE or benzene induction and biomass grown in peptone without induction (control) were used for FT-IR spectroscopy. Before analysis, the samples were dried in hot air oven at 50°C for 2 hours. Pellets formed by milling samples with KBr were subjected to FT-IR analysis and performed with a Nicolet impact 400 FT-IR spectrophotometer fitted with a high-linearity room temperature mid-infrared detector (MIR) cooled with liquid N_2_ at the Instrumentation facility of Indian Association for Cultivation of Science, Kolkata, India. The spectra were recorded in the scan range 6000–500 cm^−1^ with a resolution of 4 cm^−1^. The IR spectra obtained were analysed with Origin Pro 8 software (OriginLab, Northampton, MA).

## 3. Results and Discussion

### 3.1. Optimisation of Conditions for Enzymatic Activity

**Figure 3:**
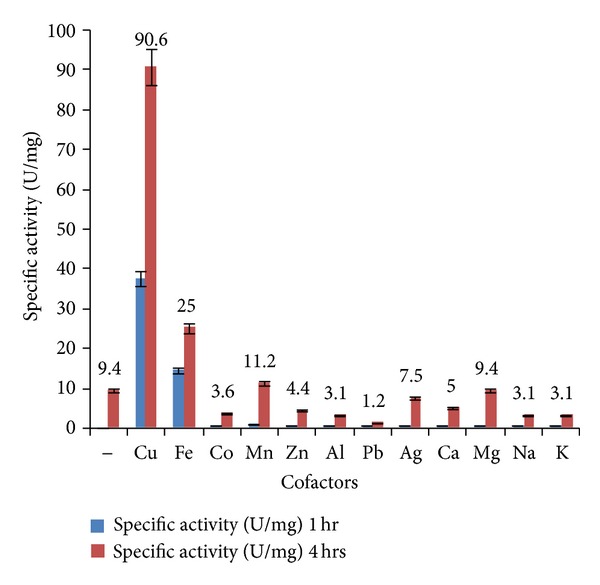
Effect of metal cofactors (1 mM) on enzyme activity in cell supernatant. Copper was found to considerably enhance enzymatic activity in comparison to other metal cofactors. Iron and manganese also have some inducing effect. Error bars with 5% SEM are displayed.

1000 *μ*L supernatant (0.016 mg extracellular proteins) was used for characterisation of enzyme activity.  Figure 3 shows the effect of 1 mM of different metal cofactors on enzymatic activity plotted with respect to specific activity (U/mg). Copper was found to enhance oxygenase activity by 10.3-fold in comparison to other metals. Iron and manganese also increased enzyme activity to some extent. Optimum copper concentration was found to be 0.7 mM. NADH concentration as a coenzyme in enzymatic reaction was also optimized, as shown in Table 1. A further increment of 14.96-fold in enzymatic activity was seen in presence of 0.05 mM NADH in addition to 0.7 mM Cu. A methane monooxygenase from *Methylocystis sp*. strain M has been reported to have a copper-active site that turned it into a powerful copper oxidase [30]. Manganese and iron were also found to enhance the PM102 oxygenase activity to some extent, indicating that Mn/Fe may play a role as an enzyme cofactor in absence of Cu. There are reports on polysaccharide oxygenases using Mn and Cu as cofactors [31, 32]. 

**Figure 4:**
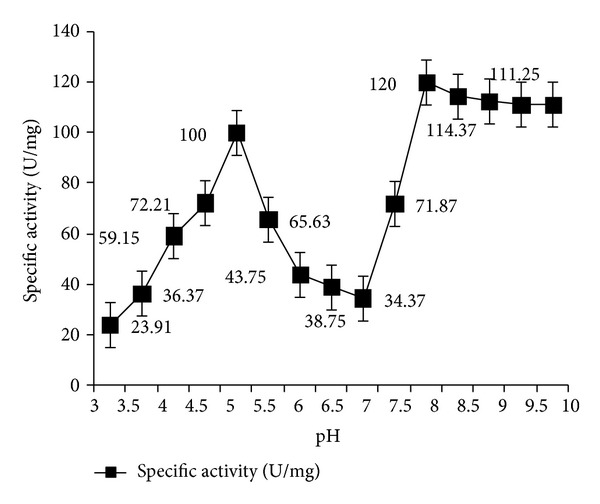
Oxygenase activity under varying pH with 0.7 mM Cu at 36°C. Optimum activity could be detected at pH 5 and pH 8, respectively. Error bars with 5% SEM are displayed.

Table 1 is listed with the effect of substrates, temperature, pH, and optimum cofactor and coenzyme concentrations of the enzyme taken from culture supernatant. Benzene was found to enhance enzymatic activity greater than TCE or any other substrate as evident from the enzymatic rate calculations: *V*_max⁡_ for benzene was 340 U/mg/min whereas *V*_max⁡_ for TCE was 170 U/mg/min. *K*_*m*_ (benzene) was 3.8 mM, and *K*_*m*_ (TCE) was 2.1 mM, respectively. Two optimum pH for enzymatic activity were noted at pH 5 (acidic) and pH 8 (alkaline), respectively.  Figure 4 clearly shows that first, there is a sharp increase in enzyme activity at pH 5 which drops around neutral pH followed by a peak at pH 8 that gradually falls into a plateau. The most probable explanation was the presence of two isoforms that was further confirmed by zymogram assay with the purified enzyme extract. Two brown bands in close proximity were detected in the zymogram that corresponded to two bands in the 70% purified enzyme fraction. 

### 3.2. Comparison of Enzyme Activity in Extracellular versus Intracellular Extracts

**Figure 5:**
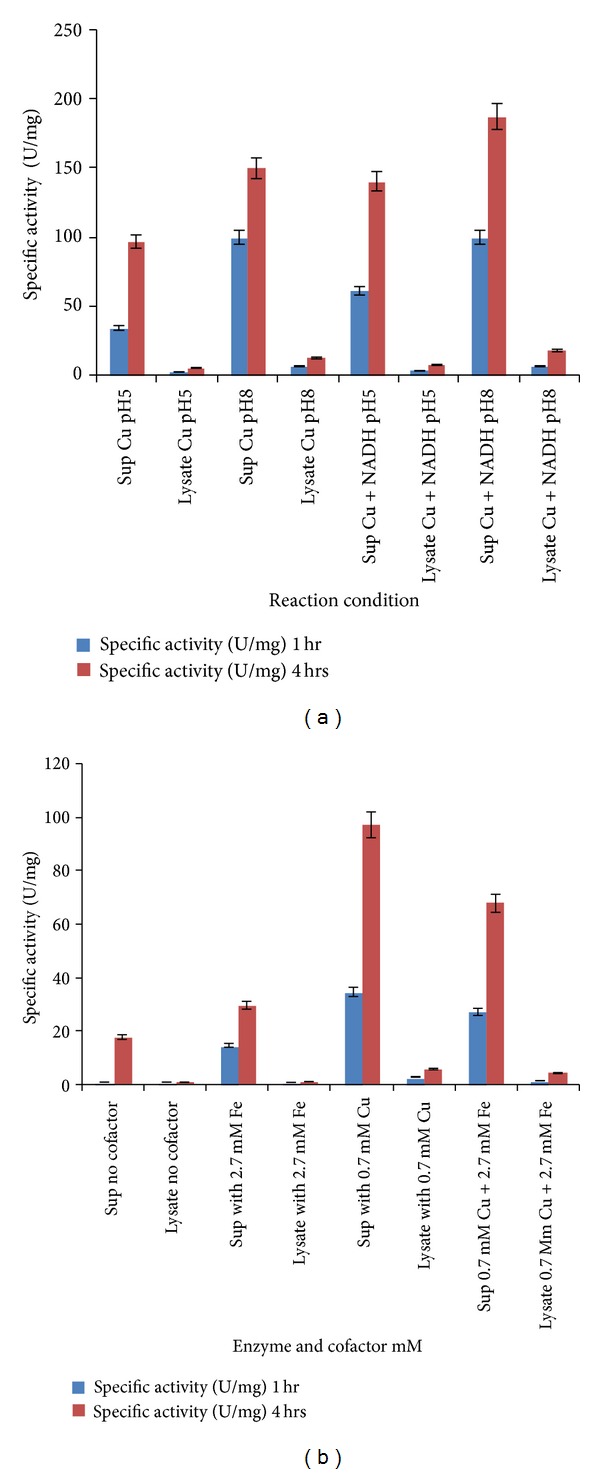
(a) Enzyme activity is maximum in culture supernatant. Comparatively, less activity is seen in cell lysate at pH 5 and pH 8. Error bars with standard error are shown. (b) At constant pH 5, enzyme activity is enhanced in presence of 0.7 mM Cu and 2.7 mM Fe. Maximum activity is seen in extracellular form. Activity in presence of both Cu and Fe is lower than that in presence of only Cu. Error bars with standard error are displayed.

All the optimisations in enzyme activity were done with 1 mL of culture supernatant (obtained from a total of 200 mL cell culture) that contained approximately 0.016 mg protein as measured by Bradford assay. To compare the enzyme activity in the intracellular fraction obtained by lysozyme extraction of the 200 mL cell pellet, the reaction was carried out under optimised conditions, that is, 0.7 mM Cu, 0.05 mM NADH, and 36°C, and at pH 5 and pH 8, respectively, as shown in Figure 5(a).  Figure 5(b) shows enzyme activity in supernatant and cell lysate at pH 5 with Cu and Fe as cofactors and without any cofactor. Oxygenase activity was found to mainly lie in the extracellular fraction. Percentage of total activity in 1 mL supernatant (0.016 mg crude protein) with 0.7 mM Cu after 4 hours was 94.5%, while activity in 100 *μ*L cell lysate (0.3 mg crude protein) with 0.7 mM Cu after 4 hours was 5.5%. Thus, percentage of total activity was maximum in the extracellular supernatant with comparatively less activity in intracellular lysate, at pH 5 and pH 8.

### 3.3. Zymogram Analysis

**Figure 6:**
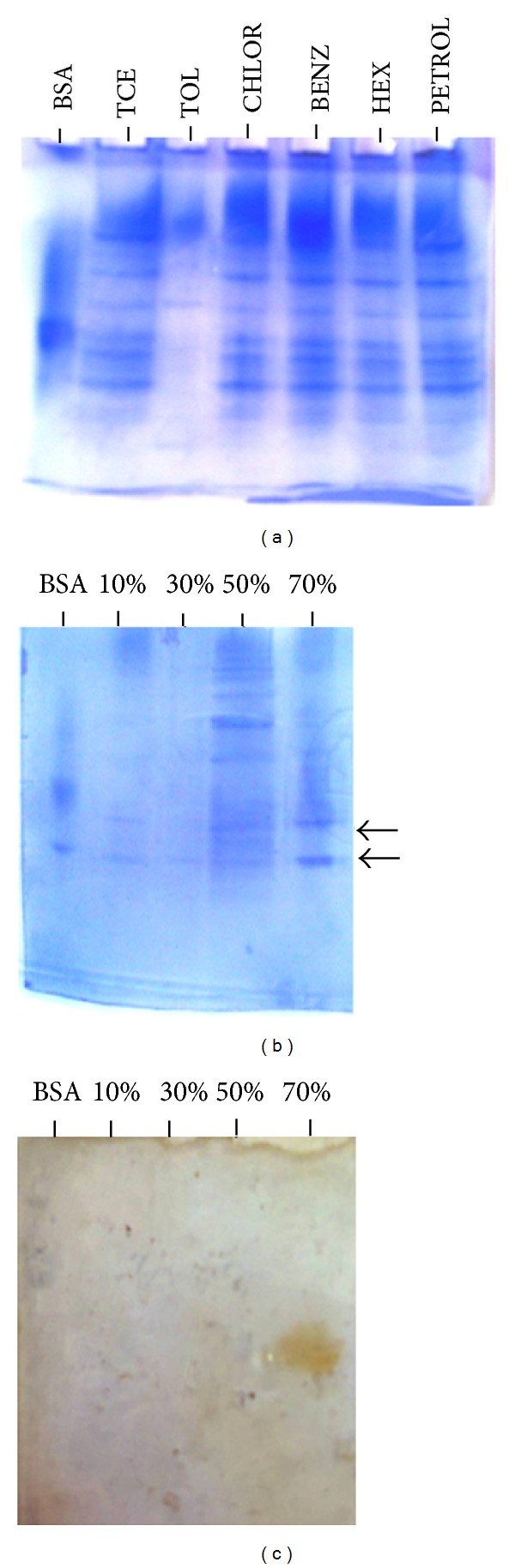
(a) The 8% native PAGE with proteins obtained by lysozyme extraction from PM102 cells grown in minimal medium with 0.2% TCE for 24 hours followed by induction with 0.2% of various carbon sources: TCE, toluene, chloroform, benzene, hexane, and petroleum for another 24 hours. (b) The 8% native gel with protein fractions purified from culture supernatant. As enzyme activity was higher in the supernatant, extracellular proteins were purified by ammonium sulphate precipitation. At 70% ammonium sulphate, the two protein bands that gave activity bands are shown with arrows. (c) The 8% native gel zymogram showing two activity bands lying in close proximity indicating two isoforms of the enzyme.

Three 8% native gels were electrophoresed under same conditions. One gel was subjected to the usual staining (Coomassie blue R250) and destaining (30% methanol with 7.5% acetic acid) process (Figure 6(a)), while the other two parts of identical gels were subjected to activity staining. One of the zymogram gels was incubated in benzene as substrate while the other zymogram was incubated in TCE as substrate. A broad brown activity band appeared only in the benzene lane that was much prominent when the gel was incubated in benzene as substrate. A faint activity response was seen when the gel was incubated in TCE as the substrate. This observation led us to believe that the PM102 oxygenase may be a cometabolic enzyme lying in the common pathway of benzene and TCE degradation. To locate the particular protein band that showed up in the activity stain, purification of the crude enzyme extract was done. As enzyme activity was considerably higher in the extracellular form, the culture supernatant was subjected to ammonium sulphate precipitation and dialysis.

### 3.4. Activity Staining with Partially Purified Enzyme Extract

Figure 6(b) shows the 8% native gel with the purified enzyme fractions. At 10% and 30%, negligible amount of protein was precipitated. Most of proteins were salted out at 50%, while at 70%, moderate amount of protein was precipitated. Specific activity with the unpurified culture supernatant was 34.37 U/mg after 1 hour, while 98.69 U/mg activity was recorded with the 70% purified enzyme fraction (2.8-fold of purification could be achieved). One U of enzyme activity is defined as the amount of the enzyme that catalyzes the conversion of 1 micro mole of substrate per minute.

Figure 6(c) depicts the zymogram with the purified enzyme fractions from the culture supernatant. It was clearly visible that two brown bands in close proximity developed in the activity stain corresponding to the two closely lying protein bands in the 70% purified enzyme fraction. Further purification of the enzyme for subsequent crystal structure prediction needs to be investigated. 

### 3.5. Fujiwara Test to Determine TCE Degradation Role of the Purified Enzyme Extracts

**Figure 7:**
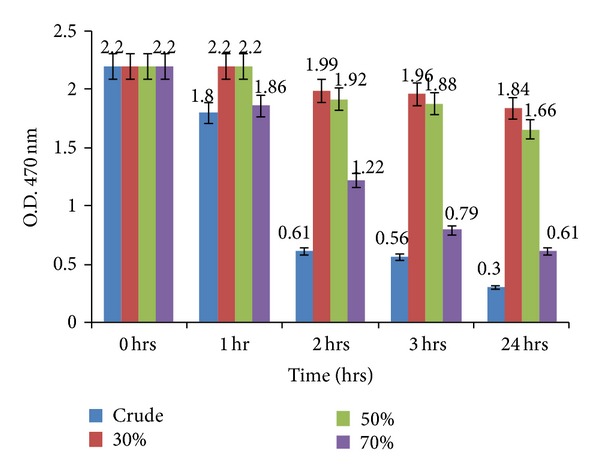
Fujiwara test showing TCE degradation by crude enzyme (culture supernatant obtained from PM102 isolate grown in 0.2% TCE with 0.2% benzene induction) and by ammonium sulphate precipitated fractions at 30%, 50%, and 70%. The 70% enzyme fraction showed comparatively higher rate of TCE degradation than the other fractions. Error bars with 5% SEM are displayed.

Figure 7 shows TCE degradation by the crude protein extract from PM102 isolate in comparison to the individual enzyme fractions obtained by salt precipitation. Higher rate of TCE degradation was seen in the 70% enzyme fraction that also reacted in activity staining. This further validates the assumption that common enzymes are involved in the degradation of TCE and benzene.

### 3.6. FT-IR Analysis

**Figure 8:**
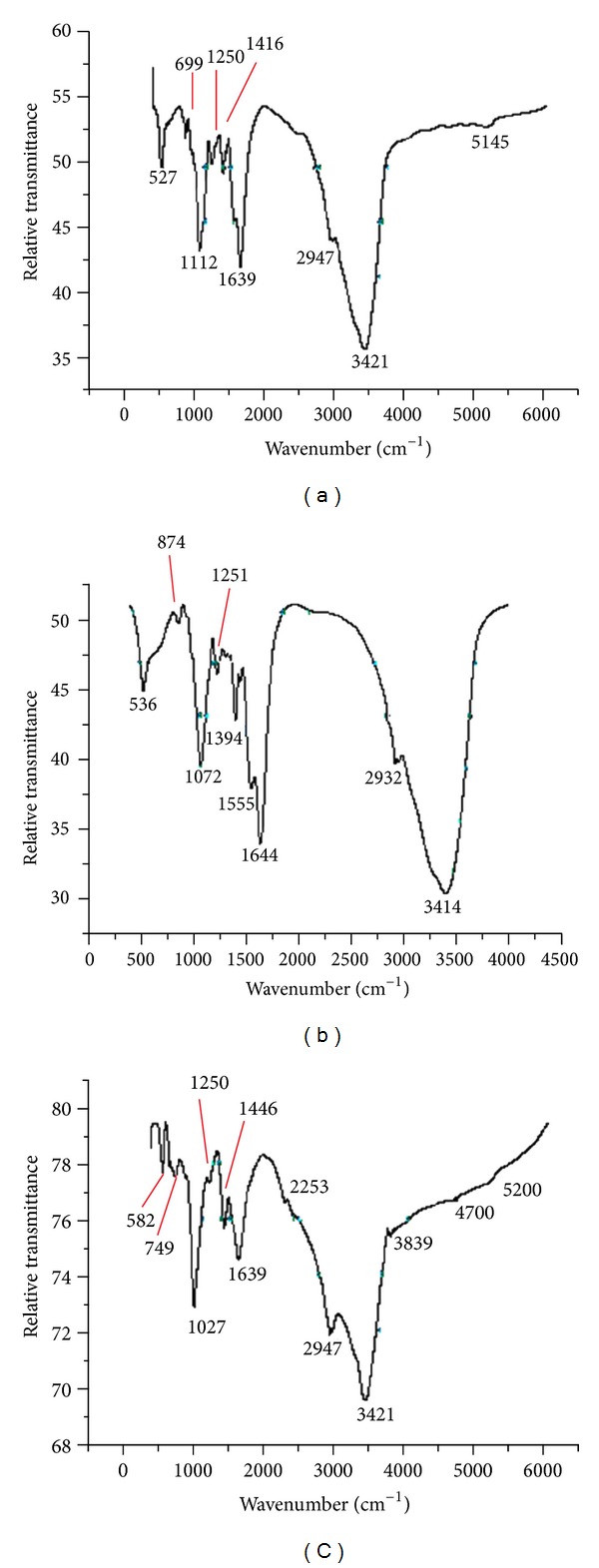
(a) FT-IR spectrum of peptone-grown PM102 biomass (control). (b) FT-IR spectra of TCE-induced PM102 biomass (treated). (c) FT-IR spectra of benzene-induced PM102 biomass (treated).

The FT-IR spectra for treated (TCE-induced and benzene-induced biomasses) and control (peptone grown biomass) are shown in Figures 8(a), 8(b), and 8(c).  Table 2 shows the identified functional groups present in the biotransformed product of TCE-treated and benzene-treated biomasses against peptone-grown biomass. Peaks at 874, 1394, and 1555 cm^−1^ corresponding to epoxy ring, tertiary alcohol and carboxylic acid were present in TCE-treated samples only. In control, peaks at 699 and 1416 cm^−1^ correspond to C–Cl stretch and C–H in plane bend. This evidence points towards the transformation of TCE by the PM102 isolate to TCE epoxide. Previous reports show a soluble methane monooxygenase from *Methylosinus trichosporium* OB3b converts TCE to chloral hydrate, trichloroacetate, trichloroethanol, and CO_2_. Toluene monooxygenase from *Burkholderia cepacia* G4 has been known to convert TCE to TCE epoxide [33]. Thus, the oxygenase involved in TCE degradation characterised in this paper is a monooxygenase as evidence of epoxide formation has been documented. Benzene-treated biomass gave peak at 749 cm^−1^ that corresponds to 1,2-disubstitution (ortho) in aromatic ring which is indicative of benzene-cis-diol or di-hydroxy benzene. Peaks at 1027, 1446, and 2253 cm^−1^ for benzene-treated cells could be designated as cyclohexane ring, aromatic ring stretch, and isocyanate, respectively.

 Isocyanates are known to have specific industrial importance in the manufacture of polyurethanes which have wide known applications in preparation of dental materials, contact lenses, and medical adsorbents and as an ingredient in automobile paints. Medical and industrial importance of cis-diols has been stated previously. Industrial importance of ethylene oxide (epoxide) can be determined from the fact that it is one of the most important raw materials used in the large-scale chemical production, mainly of ethylene glycol that accounts for up to 75% of global consumption. Ethylene glycol is used as antifreeze, in the production of polyester and polyethylene terephthalate (PET—raw material for plastic bottles), liquid coolants, and solvents. Polyethylene glycols are used in perfumes, cosmetics, pharmaceuticals, lubricants, paint thinners, and plasticizers. Ethylene glycol ethers are part of brake fluids, soaps, detergents, solvents, lacquers and paints [34]. Ethylene oxide is one of the most commonly used sterilization methods in the healthcare industry and for processing of storage facilities (tobacco, packages of grain, sacks of rice, etc.), clothing, furs, and valuable documents [35]. Thus, TCE and benzene degradation products by the PM102 isolate are of considerable significance in medicine and industrial applications.

## Figures and Tables

**Table 1:** Characterisation of monooxygenase activity from *Stenotrophomonas maltophilia* PM102.

Conditions for enzyme activity	Enzyme parameters	Specific activity (U/mg) (4 hrs)
Metal cofactors (1 mM)	Cu, Fe, and Mn	90.6, 25, and 11.2
Optimum metal cofactor	Cu	
Range of copper concentration tested	0.05 mM–5 Mm	
Optimum Cu concentration	0.7 mM	96.87
Range of NADH concentration	0.01 mM–0.2 mM	
Optimum NADH concentration	0.05 mM	140.625
Range of temperature	4°C–42°C	
Optimum temperature	36°C	103.13
Range of pH	3–10	
Optimum pH	5 and 8	100 and 120
Substrates	TCE, benzene, toluene, chloroform, hexane, and petroleum benzine	87.5, 103.75, 31.87, 40.63, 13.75, and 11.25
Optimum substrate	Benzene	103.75
Rate when benzene is the substrate	*V*_max⁡_ Benzene	340 U/mg/min
Rate when TCE is the substrate	*V*_max⁡_ TCE	170 U/mg/min
*K*_*m*_ when benzene is the substrate	*K*_*m*_ Benzene	3.8 mM
*K*_*m*_ when TCE is the substrate	*K*_*m*_ TCE	2.1 mM

**Table 2:** Interpretation of FT-IR data of TCE-treated, benzene-treated, and peptone-grown (control) PM102 biomasses. Functional groups were assigned to respective wavenumbers with reference to Coates (2000) [27].

Group frequency (cm^−1^)	Functional group
Treated (TCE-induced biomass)	
536	Aliphatic bromo C–Br stretch
874	**Epoxy ring**
1072	Cyclic ethers C–O stretch
1251	Skeletal C–C vibrations
1394	**Tertiary alcohol OH bend**
1555	**Carboxylic acid**
1644	Amide or secondary amine NH bend
2932	Methylene stretch
3414	Hydroxy group H bonded OH stretch
Control (peptone-grown biomass)	
527	Aliphatic bromo C–Br stretch
699	**Aliphatic chloro C–Cl stretch**
1112	Cyclic ethers C–O stretch
1250	Skeletal C–C vibrations
1416	**Vinyl C–H in plane bend**
1639	Amide or secondary amine NH bend
2947	Methyl C–H asym./sym. stretch
3421	Hydroxy group H bonded OH stretch
Treated (benzene-treated biomass)	
582	Aliphatic bromo C–Br stretch
749	**1,2-Disubstitution (ortho) aromatic ring**
1027	**Cyclohexane ring vibrations**
1250	Skeletal C–C vibrations
1446	**Aromatic ring stretch**
1639	Amide or secondary amine NH bend
2253	**Isocyanate (–N=C=O asym. stretch)**
2947	Methyl C–H asym./sym. stretch
3421	Hydroxy group H bonded OH stretch

## References

[1] Gibson DT, Cardini GE, Maseles FC, Kallio RE (1970). Incorporation of Oxygen-18 into benzene by *Pseudomonas putida*. *Biochemistry*.

[2] Tao Y, Fishman A, Bentley WE, Wood TK (2004). Oxidation of benzene to phenol, catechol, and 1,2,3-trihydroxybenzene by toluene 4-monooxygenase of Pseudomonas mendocina KR1 and toluene 3-monooxygenase of Ralstonia pickettii PKO1. *Applied and Environmental Microbiology*.

[3] Boyd DR, Sheldrake GN (1998). The dioxygenase-catalysed formation of vicinal cis-diols. *Natural Product Reports*.

[4] Widdowson DA, Ribbons DW (1990). The use of substituted cyclohexadiene diols as versatile chiral synthons. *Janssen Chimica*.

[5] Sheldrake GN, Collins AN, Sheldrake GN, Crosby J (1992). Biologically derived arene cis-dihydrodiols as synthetic building blocks. *Chirality in Industry, the Commercial Manufacture and Application of Optically Active Compounds*.

[6] Carless HAJ (1992). The use of cyclohexa-3,5-diene-1,2-diols in enantiospecific synthesis. *Tetrahedron Asymmetry*.

[7] Zylstra GJ, Gibson DT (1989). Toluene degradation by *Pseudomonas putida* F1. Nucleotide sequence of the todC1C2BADE genes and their expression in *Escherichia coli*. *Journal of Biological Chemistry*.

[8] Byrne AM, Kukor JJ, Olsen RH (1995). Sequence analysis of the gene cluster encoding toluene-3-monooxygenase from *Pseudomonas pickettii* PKO1. *Gene*.

[9] Johnson GR, Olsen RH (1995). Nucleotide sequence analysis of genes encoding a toluene/benzene-2- monooxygenase from *Pseudomonas sp.* strain JS150. *Applied and Environmental Microbiology*.

[10] Anderson JE, Mccarty PL (1997). Transformation yields of chlorinated ethenes by a methanotrophic mixed culture expressing particulate methane monooxygenase. *Applied and Environmental Microbiology*.

[11] Wackett LP, Brusseau GA, Householder SR, Hanson RS (1989). Survey of microbial oxygenases: trichloroethylene degradation by propane-oxidizing bacteria. *Applied and Environmental Microbiology*.

[12] Arciero D, Vannelli T, Logan M, Hooper AB (1989). Degradation of trichloroethylene by the ammonia-oxidizing bacterium *Nitrosomonas europaea*. *Biochemical and Biophysical Research Communications*.

[13] Werlen C, Kahler H-PE, van der Meer JR (1996). The broad substrate chlorobenzene dioxygenase and cis-chlorobenzene dihydrodiol dehydrogenase of *Pseudomonas* sp. strain P51 are linked evolutionarily to the enzymes for benzene and toluene degradation. *Journal of Biological Chemistry*.

[14] Mukherjee P, Roy P (2013). Cloning, sequencing and expression of novel trichloroethylene degradation genes from *Stenotrophomonas maltophilia* PM102: a case of gene duplication. *Journal of Bioremediation & Biodegradation*.

[15] Urlacher VB, Lutz-Wahl S, Schmid RD (2004). Microbial P450 enzymes in biotechnology. *Applied Microbiology and Biotechnology*.

[16] Graham SE, Peterson JA (1999). How similar are P450s and what can their differences teach us?. *Archives of Biochemistry and Biophysics*.

[17] Sono M, Roach MP, Coulter ED, Dawson JH (1996). Heme-containing oxygenases. *Chemical Reviews*.

[18] van der Werf MJ (2000). Purification and characterization of a Baeyer-Villiger mono-oxygenase from *Rhodococcus erythropolis* DCL14 involved in three different monocyclic monoterpene degradation pathways. *Biochemical Journal*.

[19] McGuirl MA, Dooley DM (1999). Copper-containing oxidases. *Current Opinion in Chemical Biology*.

[20] Murreil JC, Gilbert B, McDonald IR (2000). Molecular biology and regulation of methane monooxygenase. *Archives of Microbiology*.

[21] Zazueta-Sandoval R, Novoa VZ, Jiménez HS, Ortiz RC (2003). A different method of measuring and detecting mono- and dioxygenase activities: key enzymes in hydrocarbon biodegradation. *Applied Biochemistry and Biotechnology Part A*.

[22] Huang WE, Hopper D, Goodacre R, Beckmann M, Singer A, Draper J (2006). Rapid characterization of microbial biodegradation pathways by FT-IR spectroscopy. *Journal of Microbiological Methods*.

[23] Mukherjee P, Roy P (2012). Identification and characterisation of a bacterial isolate capable of growth on trichloroethylene as the sole carbon source. *Advances in Microbiology*.

[24] Mukherjee P, Roy P (2013). Purification and identification of trichloroethylene induced proteins from *Stenotrophomonas maltophilia* PM102 by immuno-affinity-chromatography and MALDI-TOF Mass spectrometry. *SpringerPlus*.

[25] Mukherjee P, Roy P (2013). Persistent organic pollutants induced protein expression and immunocrossreactivity by *Stenotrophomonas maltophilia* PM102: a prospective bioremediating candidate. *BioMed Research International*.

[26] Bradford MM (1976). A rapid and sensitive method for the quantitation of microgram quantities of protein utilizing the principle of protein dye binding. *Analytical Biochemistry*.

[27] Coates J (2000). *Interpretation of Infrared Spectra. Encyclopedia of Analytical Chemistry*.

[28] Coligan JE, Dunn BM, Speicher DW, Wingfield PT, Ploegh HL (1998). *Current Protocols in Protein Science*.

[29] Moss MS, Rylance HJ (1966). The Fujiwara reaction: some observations on the mechanism. *Nature*.

[30] Smith SM, Rawat S, Telser J, Hoffman BM, Stemmler TL, Rosenzweig AC (2011). Crystal structure and characterization of particulate methane monooxygenase from Methylocystis species strain M. *Biochemistry*.

[31] Hemsworth GR, Taylor EJ, Kim RQ (2013). The copper active site of CBM33 polysaccharide oxygenases. *Journal of the American Chemical Society*.

[32] Westereng B, Ishida T, Vaaje-Kolstad G (2011). The putative endoglucanase pcGH61D from phanerochaete chrysosporium is a metal-dependent oxidative enzyme that cleaves cellulose. *PLoS ONE*.

[33] Whittaker M, Monroe D, Oh DJ, Anderson S Trichloroethylene pathway map. http://umbbd.ethz.ch/tce/tce_image_map.html.

[34] Ethylene oxide product overview.

[35] Ethylene oxide.

